# Spatio-Temporal Characteristics of Global Warming in the Tibetan Plateau during the Last 50 Years Based on a Generalised Temperature Zone - Elevation Model

**DOI:** 10.1371/journal.pone.0060044

**Published:** 2013-04-02

**Authors:** Yanqiang Wei, Yiping Fang

**Affiliations:** 1 Institute of Mountain Hazards and Environment, Chinese Academy of Sciences, Chengdu, Sichuan, P.R. China; 2 University of Chinese Academy of Sciences, Beijing, P.R. China; Plymouth University, United Kingdom

## Abstract

Temperature is one of the primary factors influencing the climate and ecosystem, and examining its change and fluctuation could elucidate the formation of novel climate patterns and trends. In this study, we constructed a generalised temperature zone elevation model (GTEM) to assess the trends of climate change and temporal-spatial differences in the Tibetan Plateau (TP) using the annual and monthly mean temperatures from 1961–2010 at 144 meteorological stations in and near the TP. The results showed the following: (1) The TP has undergone robust warming over the study period, and the warming rate was 0.318°C/decade. The warming has accelerated during recent decades, especially in the last 20 years, and the warming has been most significant in the winter months, followed by the spring, autumn and summer seasons. (2) Spatially, the zones that became significantly smaller were the temperature zones of −6°C and −4°C, and these have decreased 499.44 and 454.26 thousand sq km from 1961 to 2010 at average rates of 25.1% and 11.7%, respectively, over every 5-year interval. These quickly shrinking zones were located in the northwestern and central TP. (3) The elevation dependency of climate warming existed in the TP during 1961–2010, but this tendency has gradually been weakening due to more rapid warming at lower elevations than in the middle and upper elevations of the TP during 1991–2010. The higher regions and some low altitude valleys of the TP were the most significantly warming regions under the same categorizing criteria. Experimental evidence shows that the GTEM is an effective method to analyse climate changes in high altitude mountainous regions.

## Introduction

Contemporary global warming has received worldwide attention as increasing numbers of climate fluctuations and catastrophes have been reported in recent years. The Intergovernmental Panel on Climate Change (IPCC) Fourth Assessment Report (AR4) indicated that warming has increased since the 1910s, that the global annual mean temperature increased by 0.74°C from 1906 to 2005, and that this temperature is likely to increase 1.1–6.4°C by 2100 [1]. In China, the temperature has increased 0.4–0.5°C from 1860 to 2005, and the increase in winter temperatures has been apparent since 1951; nineteen ‘green winters’ have been experienced since 1986/1987 [2]. Regarding global warming, the projected climate trends are emphasised by the existing literature. The accumulating evidence suggests a strong and sustained increase in the global average temperatures since the 1970s, which is superimposed on the more gradual warming of Earth over the entire 20th century, and increasing levels of uncertainties in future weather patterns.

Other representative climate indicators, e.g., snow, glaciers and permafrost of the cryosphere, particularly in high altitude mountains, have proven more sensitive and vulnerable to climate change than other land surface components. The warming climate has led to the melting and shrinking of glaciers and permafrost. As Oerlemans concluded, the worldwide extent of glacier retreat between 1884 and 1978 was proportional to the extent of global warming (0.66±0.1°C) over the same period, and the total area of the world’s mountain glaciers are predicted to decrease by 1/3–2/3 in the 21st century [3], [4], [5]. Furthermore, overall warming could lead to accelerated ice mass loss, and accelerated iceberg melting is contributing significantly to the increase in sea levels.

Unfortunately, climate changes at the three poles of Earth, especially in high altitude regions, are more severe than in middle and low altitude regions but have not received adequate attention [5], [6], [7], [8], [9]. High altitude regions do not face the direct influences and hazards that global warming presents to coastal and relatively low altitude regions (from rising sea levels); however, other subtle changes in heterogeneous systems, particularly in hydrological cycles and ecosystems, are much more significant at these elevations because of the impact on atmospheric circulation, species redistribution and ecosystem processes at different altitudes and locations and in different climate regimes. In addition to these concerns, high altitude regions often have their own unique characteristics that relate to global warming. Several studies have reported that the surface climate changes at high elevations show more pronounced warming trends than those of low altitude areas, i.e., an elevation dependency of climatic warming [8], [10], [11]. Currently, the relevant studies of temperature rise and isotherm uplift as a result of global warming in high altitude regions are mainly focused on the following areas: (1) Snowline or equilibrium line altitude (ELA) elevation with glacier shrinkage [12], [13], [14], [15]; (2) Treeline and ecotone rises toward the poles or higher altitudes in response to warming [16], [17], [18], [19], [20]; (3) Climate belt or species range shifts poleward or towards higher elevations [21], [22], [23], [24]; and (4) Land use cover change (LUCC) [9], [25], [26]. These aspects have provided a general framework for climate change in high altitude regions, but climate changes in high altitude mountains are quite different from those of flatlands because of their complicated topography conditions and vertical zonality [21], [22]. However, because of the lack of adequate observational data at the spatial and temporal resolution levels required for climate research, it has only been in the last few decades that investigations into contemporary climate change in high altitude mountains have been conducted [7], [9]. Analysis of the climate change differences in both the spatial and temporal scales of high altitude mountains, as well as detailed studies of alpine climate change, are necessary.

The Tibetan Plateau (TP) is called ‘the Antenna of the Earth,’ as it is highly sensitive to climate change, and climate indicators in this area are much more representative than those of lower altitude regions [27], [28]. Warming in the TP has been mainly characterised by an increase in temperature and stable precipitation accompanied by a decrease in the potential evaporation capacity in recent years, according to previous studies of contemporary environmental change on the plateau [29], [30], [31]. Observational data from recent Chinese investigations on glacier and permafrost degradation and its environmental effects in the TP indicate that a large portion of the TP has experienced significant warming since the mid-1950s [7], [31], [32]. The permafrost were undergoing accelerated degradation, while mountain glaciers had extensive melting period and intensifying ablation, and accelerated shrunk in the last half of the 20th century [32], [33], [34], [35]. A great deal of evidence has been obtained from the elevated snowline/ELA and treeline resulting from glacier retreat caused by temperature increases in the TP [15], [20]. The rapid changes in climate conditions, especially in the permafrost and glaciers, have already caused deterioration of the environment and ecosystem of the plateau [36], [37]. Human-induced warming and LUCC in the TP have had a more significant impact on the local and regional scales than non-anthropogenic influences [38], [39], [40]. In addition, numerical simulations indicate that the air temperature of the TP will continue to increase in the 21st century, which is projected to improve the frigid and arid environment on the plateau [7], [10], [41]. Regarding the warming trend differences, Yang et al. [42] considered the air temperature increases to be most significant in the central, eastern, and northwestern parts of the plateau. Liu et al. [43] found that the warming trends in winter night-time temperatures were among the highest when compared with the surrounding regions of the TP. After examining the spatial and temporal variations of the monthly mean temperature at 71 stations with elevations above 2000 m asl. in the eastern and central TP during 1961–2004, You et al. [29] concluded that the northeastern subregion of the TP had the most significant warming trends, especially in winter and autumn. Vertically, Liu and Chen [44] revealed a more pronounced warming pattern at high elevations based on examination of the temporal trends in monthly surface temperatures from 97 weather stations above 2000 m asl. in the eastern TP and its vicinity during 1955–1996, a conclusion that has since been confirmed by numerical experiments [10]. This tendency may continue in future climate change scenarios [8]. However, You et al. [45] found that there was no relationship between elevation and temperature extremes in the eastern and central TP. There were also no simple linear relationships between the magnitudes of elevation and temperature trends based on the 71 homogenised surface stations with elevations above 2000 m asl. in the eastern and central TP [46].

Temperature is one of the key indicators of climate and usually manifests the properties of the climate, and changes in temperature trends and spatial distribution patterns usually suggest similar changes in climate. However, analysis of temperature zone or climate zone shifts in high altitude mountains is scarce in the existing literature. The objectives of this paper are the following: (i) to establish a generalised temperature zone model for high altitude regions and use it to evaluate the trends of contemporary climate change in the TP and (ii) to examine the differences of the warming trends in the temporal and spatial dimensions. The organisation of the paper is as follows: (1) Materials and methods, in which we establish a Generalised Temperature zone Elevation Model (GTEM); (2) Results, in which we use the TP as a case study and calculate the areas of each temperature zone every 5 years and analyse the shift of the GTEM and temperature changes in both the horizontal and vertical dimensions under climate change; (3) Discussion, in which we compare our results with previous relevant studies of the TP and discuss the warming trend differences in these temporal-spatial analyses; and (4) Conclusions, in which the outcomes of this study are summarized.

## Materials and Methods

### Basic Models

#### (i) Mean troposphere lapse rate

The lapse rate is defined as the decrease rate of temperature with height for an atmospheric variable. Because the energy of the aerosphere results from long-wave radiation emitted by the Earth, temperature decreases as height increases. Thus, the adiabatic lapse rate is the rate of decrease with height and not simply the rate of change. In general, an adiabatic lapse rate is the negative of the rate of temperature change over the altitude change:
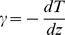
(1)Where *γ* is the lapse rate given in units of temperature divided by units of altitude, *T* is temperature, and *z* is altitude [47]. If the air is dry and the lift process is adiabatic, the rate of temperature *γ* fall is 1°C per 100 meters of lift or 5 l/2°F per 1000 feet of lift. Because air always contains some moisture, the Average Adiabatic Lapse Rate (AALR) is the mean lapse rate of the temperature and is between the dry adiabatic and the unsaturated moist adiabatic rates below the condensation level at approximately 0.65°C per 100 meters or 3.3°F per 1000 feet. Therefore in the troposphere, as the elevation increases, the temperature decreases with the AALR and in the vertical dimension and behaves as an isotherm.

#### (ii) Temperature zone Elevation Model (TEM)

**Figure 1:**
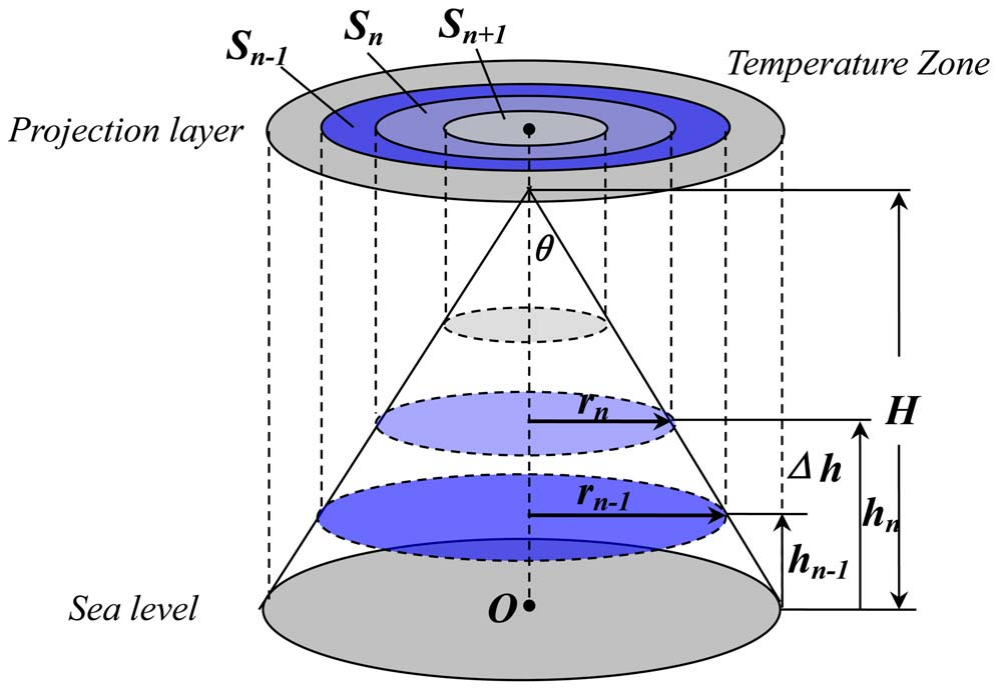
A cone-like mountain model. The elevation/temperature contours are projected on the projection layer and get a series of Temperature Zones (e.g., *S_n-1_*, *S_n_*, *S_n+1_*, …).

The relationship between the temperature zone and elevation can be informed by the AALR, and in the vertical dimension, the isotherms can be projected into the projection layer. As an alpine region usually contains a series of mountains with different shapes and sizes, the shapes of the mountains must be simplified to create a representative but manageable model. A mountain can be simplified to a circular cone shape, as shown in Fig. 1, and an alpine region can be considered to contain a series of such circular cones of different sizes.

In Fig. 1, *C_1_*, *C_2_*, *… C_n_* are a series of isotherms on the circular cone, and their areas that are projected on the projection layer are the temperature belts that need to be measured. The temperature zone can be defined as the loop between two adjacent isotherms on the projection layer. In the model, these zones are the loops *1*, *2*, *… n*. The elevation interval 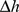 can be expressed as the absolute elevation between location *n* and location *n-1*:
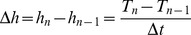
(2)


Because the vertical temperature lapse rate 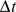 is an invariant, it remains stable in a certain location (certain longitude and latitude); here, 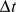 is equal to the AALR. The elevation interval 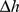 in the vertical direction is also an invariant because the isotherm interval 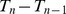 is unchanged.

To measure the areas of the temperature loops, the radii of these circles are also required. The radii of these circles are defined by 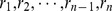. The areas of these temperature loops *S_n_* with elevation *h_n_* can be expressed as
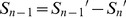
(3)

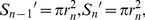
(4)


In which 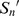 is the area of the contour’s horizontal section, and 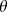 is the angle between the cone generatrix and vertical axis. Therefore,
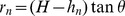


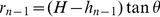
(5)


Let 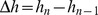 so 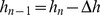. Substituting 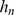 into (2)–(5) yields 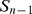,
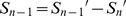


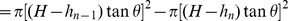


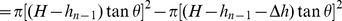


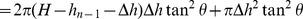



Consequently, 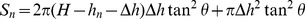


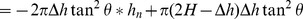


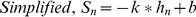
(6)


**Figure 2:**
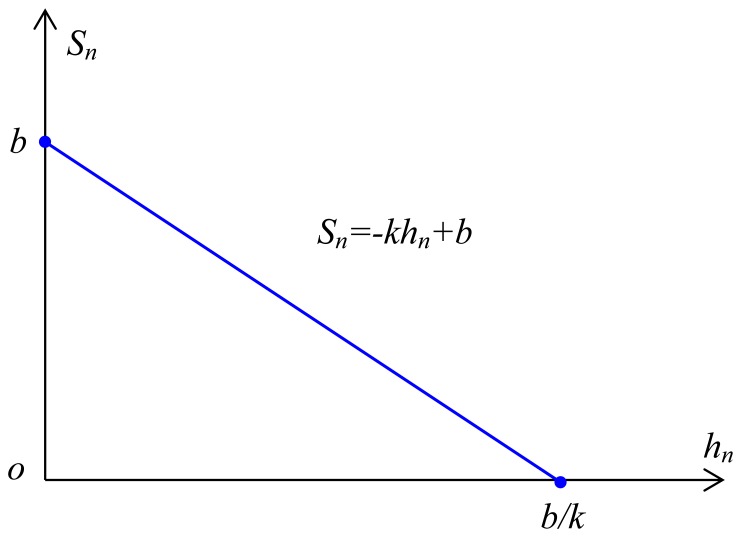
The relationship between the temperature zone (*S_n_*) and the elevation (*h_n_*) in Temperature zone - Elevation Model (TEM). Clearly, they are the linear relationship as *h_n_* increases, the area of temperature zone *S_n_* decreases correspondingly.

In which 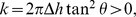 and 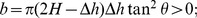 obviously, both of these are invariants. In equation (6), if 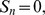 then 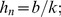 if 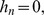 then 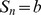. There is an obvious linear relationship between the temperature zone (*S_n_*) and elevation (*h_n_*). As the elevation *h_n_* increases, the temperature decreases, and the area of the temperature zone (*S_n_*) decreases correspondingly. The area between two adjacent isotherms on the projection layer (the temperature zone) is only directly associated with the elevation. These variables have a simple negative linear correlation in the TEM (Fig. 2).

#### (iii) Generalised Temperature zone Elevation Model (GTEM)

It is clear that the temperature zone is only dependent on the elevation and that the variables have a negative linear correlation in equation (6). In the TEM, the mountain is simplified to a circular cone and has a specified surface in which the temperature zone can be calculated by a simple linear function. However, in actuality, the elevations of mountains with relatively young ages nearly follow the normal distribution [48], [49]. This distribution may be defined as
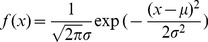
(7)


The distribution is always described as 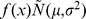, (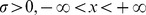). Based on (6), the function (6) may be simplified to 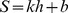
_,_ and when the independent variable *x* is substituted in (7), the distribution density function can be represented as
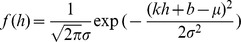



This may be transformed as
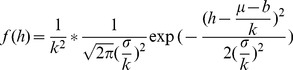
(8)


Comparing the forms of (8) and (7), 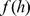 is observed to obey the normal distribution, with the mean 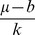, the standard deviation 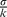 and the coefficient 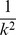, namely,
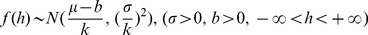
(9)


**Figure 3:**
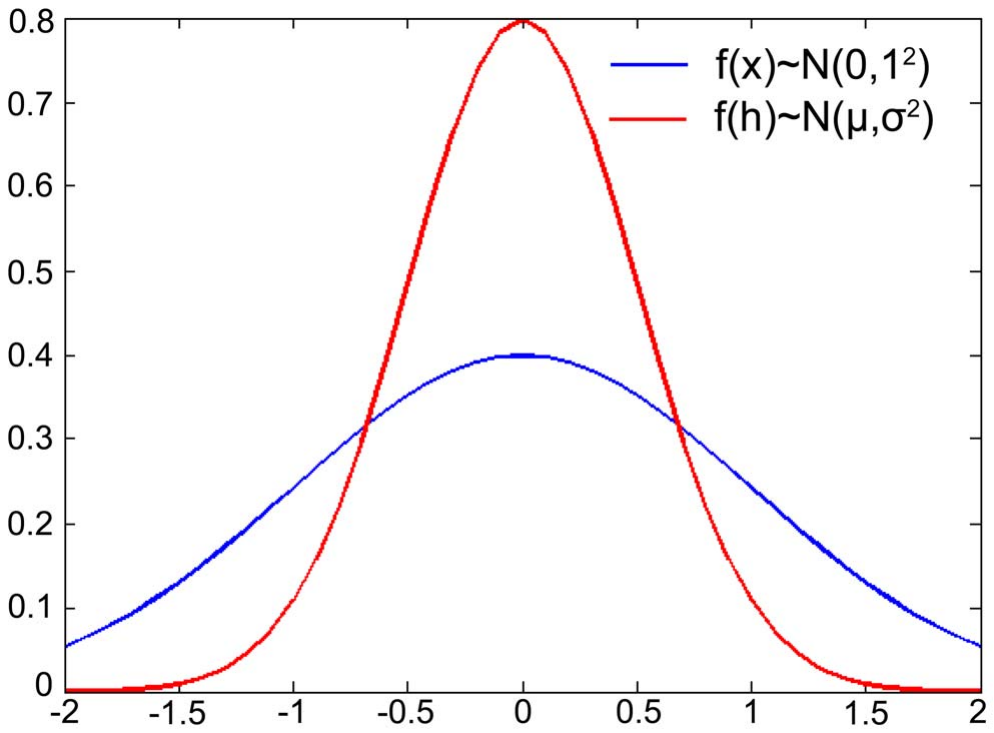
The curves of standard normal distribution *f*(*x*)∼*N*(0,1^2^) (the blue) and *f*(*h*)∼*N*(*μ*,*σ* ^2^) (the red). It is much peaked with smaller *σ* under the same *μ*.

This expression (9) demonstrates that the distribution of temperature zones also obeys a normal distribution with respect to elevation. The statistic map of temperature zones with elevation is shown in Fig. 3.

#### (iv) The shift of the GTEM under climate change

**Figure 4:**
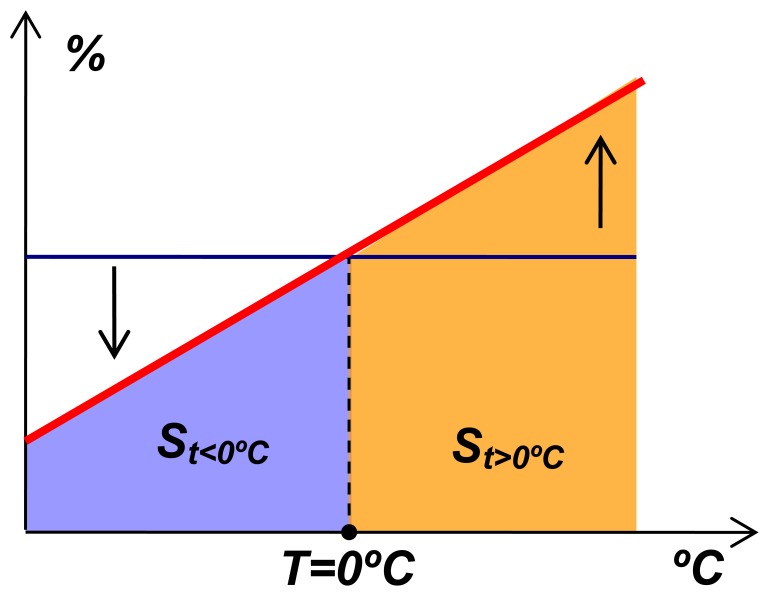
The total area of temperature zones below the Zero Isotherm (*S_t<0°C_*) shrinks and the total area of temperature zones above the Zero Isotherm (*S_t>0°C_*) increases under global warming.

**Figure 5:**
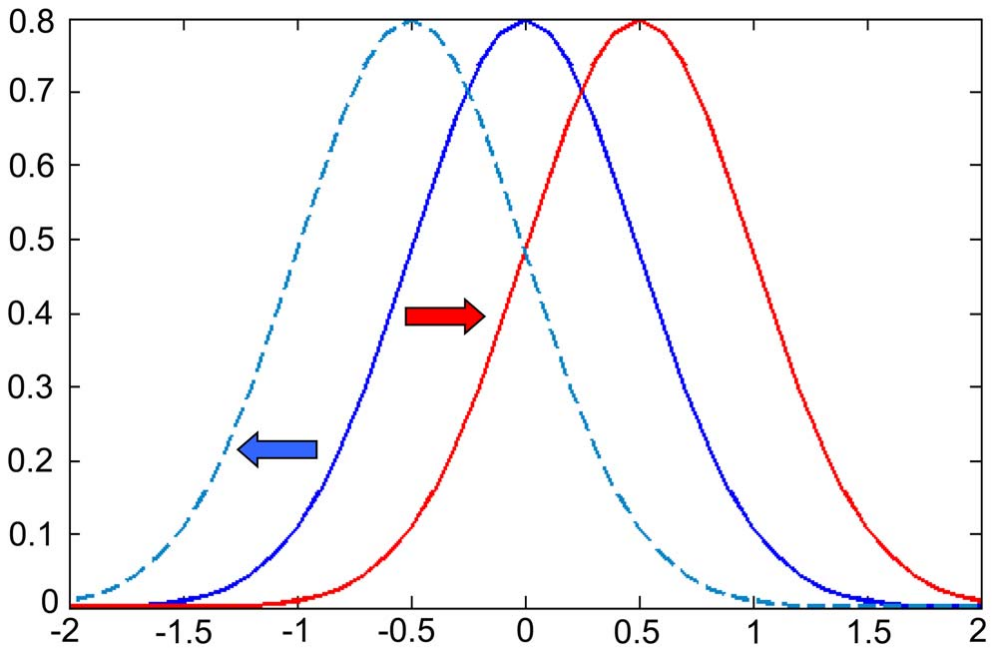
The moved curve of the normal distribution of temperature zones under climate change. If the climate is warming, then the *S_t<0°C_* getting shrunk and the *S_t>0°C_* getting raised and the curve will move towards the positive X-axis (the red), and when the climate is getting cold it will move towards the negative X-axis (the blue).

Generally, the distribution of temperature zones can be plotted, as shown in Fig. 3, and remains invariable for a period of time. However, if the climate regime changes, as occurs with rising temperatures, then the curve will move accordingly and become deformed. The isotherms become elevated due to warming over a wide spectrum of elevations in mountainous areas, especially in the plateaus. Accordingly, the area of the temperature zones with the same values will change. As a result, the total area *S_t>0°C_* of the temperature zones accumulating upwards to a particular value (e.g., 0°C towards the bottom of the mountain) is increased, and the total area *S_t<0°C_* accumulating downwards from this value (towards the top of the mountain) shrinks, or vice versa (Fig. 4). If the area of every temperature zone is plotted in the X-Y coordinate system according to expression (9), the normal distribution curve of the total area for every temperature zone will move towards the positive X-axis (the right direction) under conditions of global warming as the temperature rises. If the climate is cooling, then the curve will move in the opposite direction towards the negative X-axis (the left direction, Fig. 5).

Therefore, the GTEM model can be used to judge the presence of climate warming. A simple method to detect climate change is to calculate the areas of all of the temperature zones, plot them in the coordinate system and fit them with the normal distribution curve; if the curve moved towards the right, the climate is warming, while if it is moving in the opposite direction (towards the left of the X-axis), the climate is cooling. The gap between the two adjacent curves shows the rate of change and reveals the magnitude of climate change. However, the GTEM curve will be stable in lower altitude, flat regions due to their narrow range of elevations and monotonic topographies; thus, the model is unsuitable for such areas.

### Study Area

**Figure 6:**
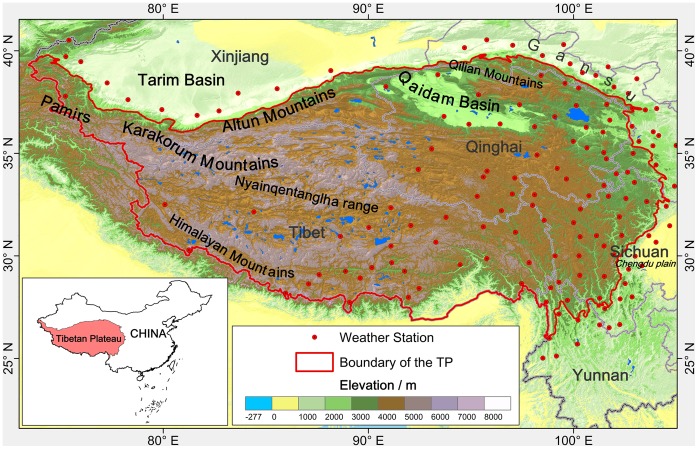
The location of the study area of the Tibetan Plateau (TP) in China.

The GTEM model should be applied in mountains or high elevation regions. If the climate is warming quickly, then a flat plain at low altitude would not show characteristics of climate change in the GTEM. If the altitude of isotherm exceeds the maximum elevation of the plain, then the temperature zone’s area, e.g., *S_t>0°C_*, will remain unchanged. Considering these requirements of the model, we chose the Tibetan Plateau (TP) as the study area. The TP is distinguished by its high altitude and cold environment and has been called the Roof of the World. After the Hungarian geologist Lóczy [50] first studied the region and defined its boundaries, the TP has become an area of international scientific observation and focused research in recent decades. However, there is not a definitive consensus on the boundaries and extent of the TP. Zhang et al. [51] have suggested that the range of the TP include the Himalayan Mountains in the south and the Kunlun Mountains, the northern margin of Altun and Qilian Mountains in the north, along with western reaches to the Pamirs and the Karakorum Mountains and eastern reaches to the Chengdu plain. In this paper, we chose the Chinese region of the TP along with the surrounding altitudes over 3500 m above sea level (asl.) as our study area. The southern boundary of the TP is the Chinese National Boundary (Fig. 6). The area of the TP is approximately 2.57 million square kilometres (sq km).

### Data Acquisition and Methods

The DEM (Digital Elevation Model) data with cell size 90 m * 90 m were downloaded from the WIST geo-database of NASA (http://reverb.echo.nasa.gov/reverb/redirect/wist, or http://srtm.csi.cgiar.org/SELECTION/inputCoord.asp) and were resampled to the resolution of 1 km * 1 km for temperature rasterization. The annual mean surface air temperature data and the monthly mean surface air temperatures were provided by the National Climate Center of China Meteorological Administration (http://cdc.cma.gov.cn/home.do). The annual temperature data were derived from the annual mean temperature records of 686 weather stations in China dating from 1961 to 2010. To calculate the temperature zone’s area, the raster temperature data with the GRID format is needed. Currently, there are several methods to get the rasterized temperature data according to the literature (e.g., [52], [53], [54], [55]). Ninyerola et al. [54] and Liao et al. [55] conclude that the Multiple Linear Regression combined with Spatial Interpolation is the minimum error approach to get the rasterized temperature data with the GRID format especially in the complex topography areas. And comparing with other interpolation methods, the Inverse Distance Weighting (IDW) is optimum for temperature interpolation [53], [54], [55]. Considering the complex topography and the uneven and very sparse distribution of the observation stations in the western part of the TP, in the paper, the Multiple Linear Regression combined with the Spatial Interpolation is used to rasterize the temperatures for reducing the rasterizing errors as much as possible. Based on this method, the temperature can be considered as the quantitative regular part *A* of environment (e.g., longitude, latitude and altitude) and the non-regular part *B* (e.g., topography, atmospheric circulation) that can not be quantified. The regular part *A* can be calculated by the regression function, and the non-regular part *B* is the *residual error*. The actual temperature is the sum of the calculated temperature *A* from the regression function and the *residual error B*. The expression is
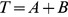
(10)


As *A* is the actual temperature regression function of latitude, longitude and altitude, the expression is changed to.
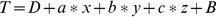
(11)


*T* (°C) is the calculated actual temperature in the meteorological station. *D* is the regression constant; *a*, *b*, *c* are the regression coefficients of the multiple linear regression function; *x* is the *Latitude* (°), *y* is the *Longitude* (°) and *z* is the *Elevation* (m asl.). *B* is the *residual error* between the calculated temperature by the regression function and the observation temperature. The regression functions of the annual mean temperatures in the 144 meteorological stations across the TP and its vicinity (160 km enlarged region, almost 1.2 degree in latitude/longitude; There are 9 incomplete records stations on the TP.) in the 5-year intervals are calculated. The *x* is the *Latitude* (°), *y* is the *Longitude* (°) and *z* is the *Elevation* (m asl.), *N* = 135, *p*<0.0001. The regression functions are listed as follows:
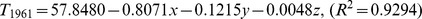


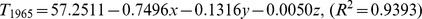


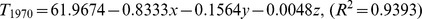


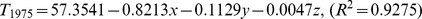


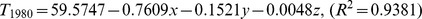


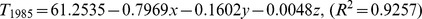


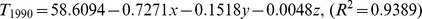


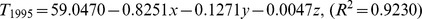


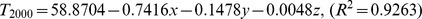


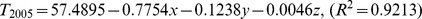


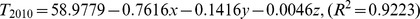



From the regressions, all of the regression functions have high correlation coefficients *R* ^2^ (all of the *R* ^2^ are above 0.92) and *P*<0.0001. The regression functions are reliable to calculate the temperatures in each station. So, the regular part *A* can be calculated by the regression functions per pixel by the DEM (Digital Elevation Model, contains the latitude, longitude and elevation in each pixel) on the whole region year by year. The *residual error B* is the difference that the calculated temperature by the regression function subtract the observation temperature in each station. After the calculation, the *residual errors* are interpolated into the whole region by the IDW with the same resolution of 1 km * 1 km as part *A*. The sum of the two parts is the rasterized temperature on the whole TP in the current year. The other years can be repeated using the same method.

**Figure 7:**
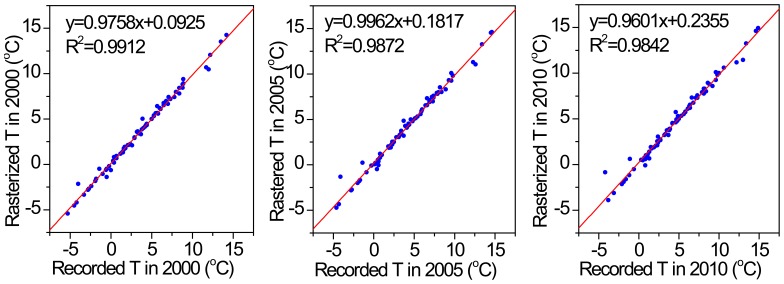
The relationship between the rasterized temperature (Rasterized T) and the observation temperature (Recorded T) in the 82 meteorological stations on the TP in 2000, 2005 and 2010. (The other years are similar to them and have not been listed).

Additionally, to calculate the area of each temperature zone, the reliability and accuracy test of the rasterized temperature data is needed. We compared the rasterized temperatures (Rasterized T) with the observation temperatures (Recorded T) in the 82 stations on the TP in 2000, 2005 and 2010 (Fig. 7, The other years are similar to them and have not been listed). The correlation coefficients *R* ^2^ are 0.9912, 0.9872 and 0.9842, respectively. The mean relative errors are 9.6%, 12.1% and 9.7%, respectively. And the mean relative errors below 5% account for 72%, 67% and 68% of the total tested stations, respectively. The tests demonstrate that the rasterized temperature data have the high accuracies and they are reliable for the area calculation.

The temperature zones are divided for every 2°C. The 1 km*1 km raster temperature data were converted into shape files to calculate the area of each zone with the same temperature. After geographic projection and clipping by the range of the TP, the area of each temperature zone (*S_n_*) was calculated. To simplify the calculations while maintaining the trends of the climate change in recent decades, the data were filtered over 5-year intervals. All of these analyses were calculated using ESRI ArcGIS 10. In the discussion section, the monthly surface mean temperature data are the original records of each meteorological station, but they have not been converted to the GRID format.

## Results

### The Dynamic of the GTEM Curve

#### Model test

**Figure 8:**
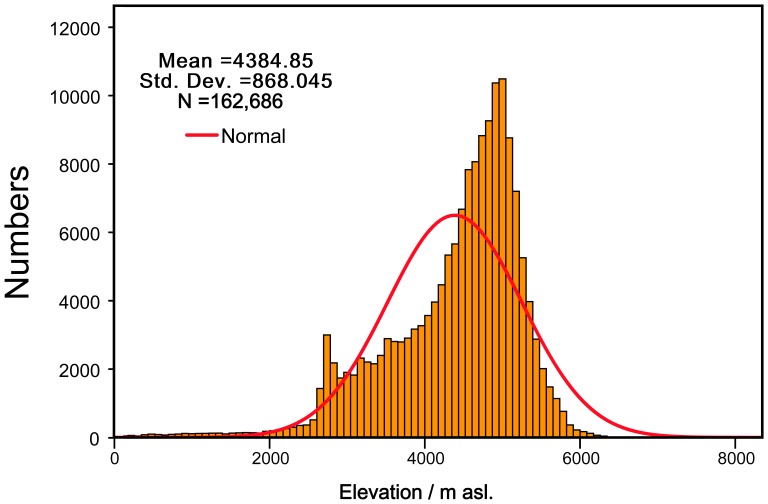
The distribution pattern of the elevations in the TP and their statistical numbers. It can be proved that it manifests a skew-normal distribution with a steep kurtosis.

The statistical results of the elevation in the study area are plotted based on the GTEM (Fig. 8). The horizontal-axis of the figure represents the elevations of the TP, and the vertical-axis of the figure represents the statistical numbers of cells with the same elevation. Approximately 80 per cent of the elevations are between 3000 to 5500 m asl., and the peak value is located at approximately 5000 m asl. This result demonstrates that most parts of the TP have high altitudes and that alpine characteristics are prominent in the region.

The elevations of the TP manifest an approximately skew-normal distribution, as shown in Fig. 8. To determine the distribution regime of the elevation, the Kolmogorov-Smirnov test (KS-test) was used in this study, as the KS-test has the advantage of making non-parametric assumptions about the distribution of data [56]. The total number of grids is 162686, the mean elevation is 4384.85 m asl. and the std. deviation of the elevation is 868.045 m. Under the KS-test, the coefficient of skewness (*Sk*) and the coefficient of kurtosis (*Ku*) were calculated. The results show that the coefficient of skewness *Sk* = −1.177<0, the coefficient of kurtosis *Ku = *1.840>0, and the 2-tailed *P = *0<0.05. Therefore, the distribution pattern of elevation in the TP shows a negative skew-normal distribution with a steep kurtosis of 1.840. Azzalini [48] and Mudholkar et al. [49] have proven that the skew-normal distribution has an equal density function after it is reparameterised using a skewness parameter *ε*. Accordingly, the function (7) is proper for this study, and the GTEM is correct for modelling the distribution of temperature zones.

#### The forward moving of the GTEM curve

**Figure 9:**
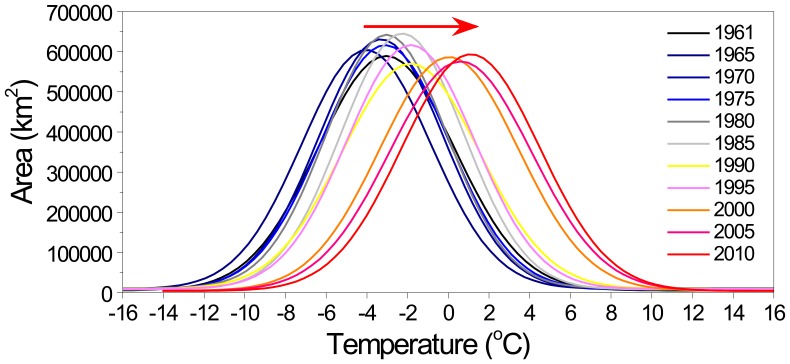
The distributions and changes of the 2°C interval annual mean temperature zones in the characteristic years.

The shift scenarios of the GTEM under climate change are discussed in Fig. 5. To examine the trends of climate change in the TP over the past 50 years, the 2°C interval annual mean temperature zones for every 5 years were calculated and fitted by Gauss Curve. The statistical results are shown in Fig. 9.

**Figure 10:**
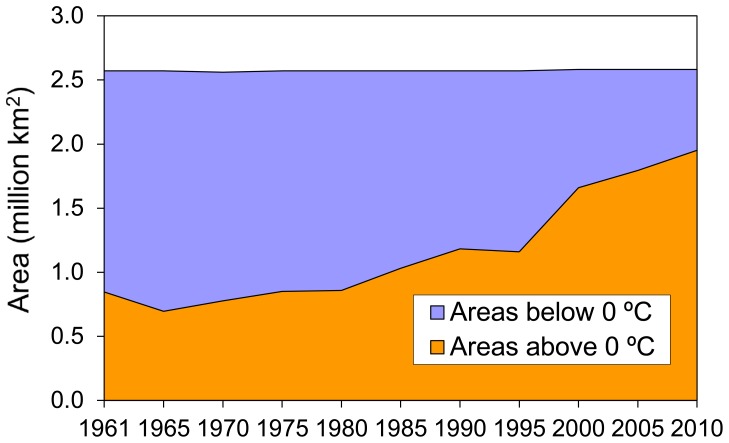
The changes of temperature zones in the TP in the past 50 years classified by 0°C annual mean temperature zone. The areas below 0°C (on the top of the TP) were getting shrunk, whereas the areas above 0°C (on the bottom of the TP) were getting extended over the study period.

In the illustration of the distribution of 2°C interval annual mean temperature zones, the curves of the earlier years are coloured in shades of navy blue, while those of recent years are coloured in shades of red. From these distribution curves, several obvious conclusions can be drawn:

1. Representative alpine characteristics. The distribution characteristics of the temperature zones show that the TP is frigid and cold, and the surface annual mean temperatures in most parts of the TP are below 0°C. When compared with low altitude areas at the same latitudes, the TP has distinct alpine cold climate characteristics. Areas with an average temperature below 0°C comprised up to 68.7 per cent of the study region from 1961 to 1980, although this ratio has decreased gradually in recent years. Because the snow lines in the TP are generally between 4200 to 5200 m asl. [57], [58], [59], most parts of the TP were in permafrost zones, and above a certain altitude, they were also covered by glaciers and permanent snow.
2. The distributions of the temperature zones are concentrated in the central peaks of the curves. In the bell curves of the distribution series, the areas of temperature zones between −10 to 8°C accounted for 92.4 per cent of the total. The summits were located in the range from −6 to 2°C. These high concentrations of low temperature zones revealed that most parts of the TP were considered to be in cold conditions and that the peaks and extremely low altitude valley regions accounted for a small proportion of the overall area.
3. The curve is moving forward. Although there exist small fluctuations in the GTEM curves, the trend of the curve has moved forward gradually over the last 50 years based on the distributions of the temperature zones in Fig. 9. The direction of the red arrow in the diagram indicates the moving tendency of the temperature zone for this period. The forward moving trend demonstrates that the climate in the TP has gradually warmed from 1961 to 2010, as suggested by the hypothesis and scenario analyses in Fig. 5. This robust warming was manifested by elevation of the isotherms in the vertical dimension, and the isotherm projections on the projection layer were changed accordingly. The changes are manifested by the enlargement of the cumulative temperature zones above a certain level (e.g., 0°C) and the shrinking of the cumulative temperature zones below this level (Fig. 10). With these changes in temperature zones, their distributions also changed.
4. Warming has accelerated, especially in the last 20 years. From the moving trend of the curves, the increasing gaps between adjacent curves illustrate that the curve is accelerating and that warming has become more severe, particularly after 1990. Although in 1995 the rate of warming retreated to levels comparable with the overall trend of the GTEM curves, the whole tendency of the curve is moving in the warming direction, and the gradually enlarged gaps between adjacent curves demonstrate that this movement is accelerating. These results show the global warming present in the TP and demonstrate that the GTEM is a sensitive and effective tool to detect climate changes in alpine regions.

### The Spatial Changes of Temperature Zones

#### Horizontal changes

Because spatial warming trends differ depending on the location, especially in regions with complicated topographies, spatial analysis of these changes is indispensable to examining the warming differences. A direct and effective method for investigating changes in the spatial dimension is to examine the changes in the areas of temperature zones.

**Figure 11:**
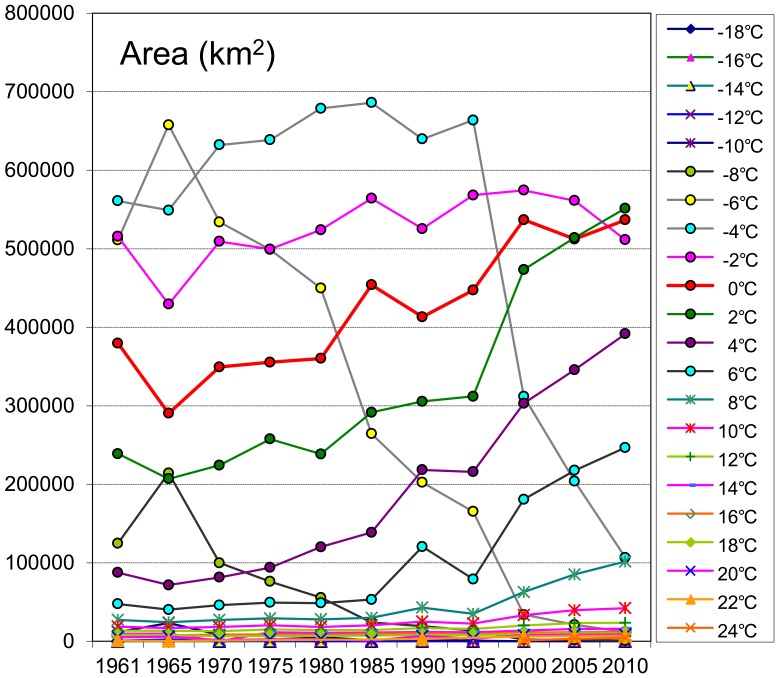
The areas of each 2°C annual mean temperature zones in every 5-year intervals.

The total areas of each 2°C annual mean temperature zone over the 5-year intervals were calculated (Fig. 11). The three most reduced temperature zones were the −6°C, −4°C, and −8°C zones. The −6°C temperature zone was the fastest zone and had decreased 499.4 thousand sq km at an average rate of 25.1% every five years from 1961 to 2010. The second most quickly shrinking temperature zone was the −4°C, which shrank 454.3 thousand sq km from 1961 to 2010 at an average rate of 11.7% every five years. During the period from 1961 to 1990, however, the −4°C zone was spreading gradually; after 1990, it began to shrink significantly. Simultaneously, however, several warmer temperature zones have been increasing in size, especially over the last 20 years. The top three most enlarged temperature zones were the 0°C, 2°C, and 4°C zones. These zones comprised a large proportion of the TP at the start of the study period, accounting for 14.8%, 9.3% and 3.4%, respectively, of the total area in 1961 but had gradually increased to 20.8%, 21.4% and 15.2%, respectively, in 2010. The average increasing rates of these zones were 4.5%, 9.9% and 17.8%, respectively, every five years.

It is obvious from the distribution curves in Fig. 11 that the most prominent changes were concentrated in certain temperature zones, especially the −6°C and −4°C zones. Low temperature zones with extremely high elevations at the roof of the TP and warmer temperature zones with low altitudes in the valley regions of the TP experienced lesser changes in comparison with other zones in the middle of the region when the climate was warming. Further analyses demonstrated that these zones only accounted for a small proportion of the area of the TP. Consequently, these small proportions meant that the regions were represented at the bottom of the diagram. In the TP, approximately 80 per cent of elevations are between 3000 to 5500 m asl., and the annual average temperatures of these regions range from −8°C to −4°C (Fig. 11). These areas account for the vast majority of the TP, with slight temperature variations, and the changes to such large areas are prominent. Therefore, the statistics indicate that these regions are more sensitive and responsive to climate warming. This pattern also explains the result that under the same absolute temperature increment, the mountainsides with gentle slopes in the GTEM are much more prone to change in the projected areas than the flatlands.

**Figure 12:**
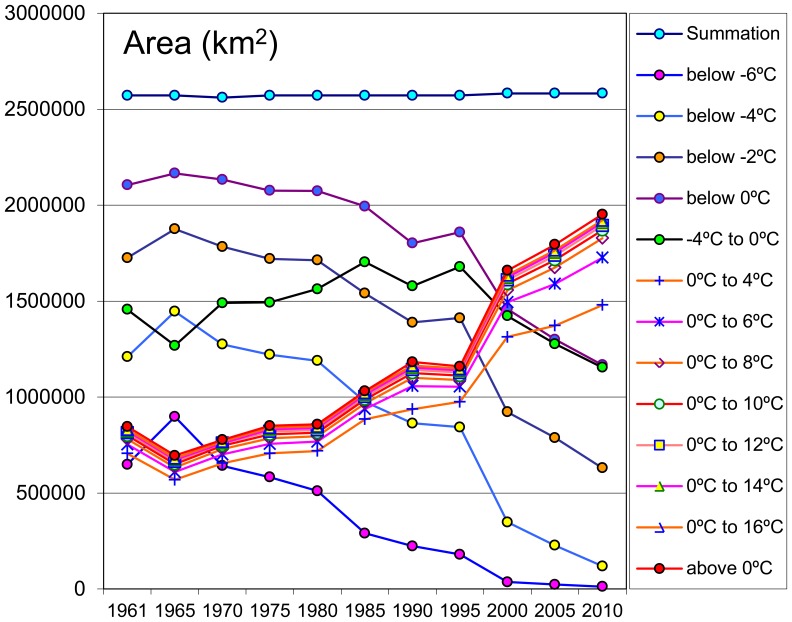
The total areas of each cumulative temperature zones within different ranges in every 5-year interval.

In addition to the changes of each individual temperature zone, the cumulative temperature zones within a certain spectrum also exhibited the same statistical warming tendencies. The trends of the cumulative temperature zones within certain scopes, i.e., the zones below −6°C, −4°C, −2°C etc., were calculated for further analyses (Fig. 12).

The calculated results revealed that four cumulative temperature zones below −6°C, −4°C, −2°C and 0°C had shrunk, while the remaining zones were enlarging simultaneously. The top three most reduced zones were the cumulative temperature zones below −6°C, −4°C and −2°C. The absolute decreases for each zone were 636.22 thousand sq km, 1.09 million sq km and 1.1 million sq km, respectively, from 1961 to 2010, which equated to decreases of 98.05%, 90.14% and 63.45%, respectively. On the whole, the cumulative temperature zones below 0°C had declined 937.64 thousand sq km from 1961 to 2010, a decrease of 44.54%. In terms of the rate, the fastest shrinking zones were the cumulative temperature zones below −6°C, followed by the −4°C, −2°C and 0°C. These statistical results demonstrate that the fastest shrinking zones were the higher elevations of the TP and that these regions were more sensitive and responsive to climate warming.

Although the total area of the zone between −4 to 0°C had expanded at the beginning of the study period, it had also begun to shrink after 1990. We doubt that the abnormality observed in 1995 is the manifestation of the complicated topography and the vibration of the 5-year interval temperature. However, each of the cumulative temperature zones above 0°C had expanded simultaneously over the past 50 years. These zones occupied almost one third of the total area of the TP, and the absolute increase in area of these cumulative temperature zones was 714.51 thousand sq km, an increase of 89.7% from 1961 to 2010 (Table S1).

**Figure 13:**
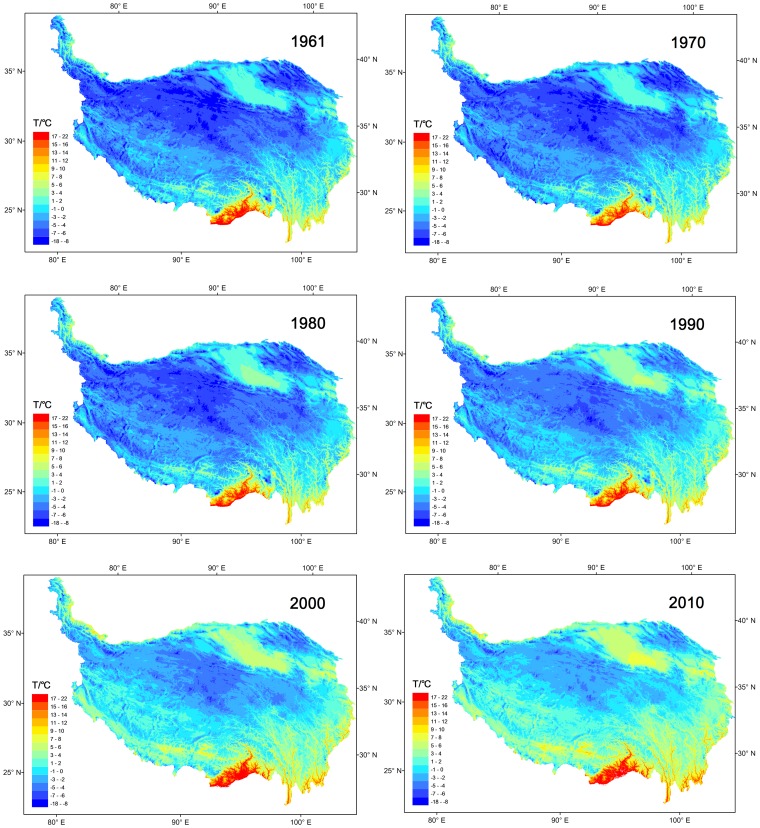
The distribution of the temperature zones in the TP in the characteristic years from 1961 to 2010 under the same categorizing criteria.

The shrinking and expanding magnitudes of each cumulative temperature zone in the horizontal dimension exhibited the trends and amplitudes of climate changes in the spatial dimension. The magnitudes and rates of change in different temperature zones varied from place to place, as have the differently accelerating rates of global warming in recent decades. On the whole, the cumulative temperature zones below −6°C in the northwestern and central TP were the most prominently reduced zones during the last 50 years. To clearly display the changes in the spatial dimension, the spatial distributions of the temperature zones were plotted in decade intervals from 1961 to 2010 (Fig. 13).

The spatial distributions of the temperature zones were nearly consistent with the elevations of the TP. The colder zones were located in the Kunlun-Hoh Xil-Tanggula Mountains, northwest of the TP, and the Himalayas-Gangdise Mountains, southwest of the TP. The warmer zones (shown in warm red colours) were located in the Brahmaputra valleys and the regions near India and Myanmar and Yunnan and the Chengdu plain, southeast of the plateau margin. In the northern TP, the vicinity of the Qaidam Basin and Qinghai Lake had relatively low altitudes of approximately 2800–3200 m asl. and was another warmer temperature zone.

**Figure 14:**
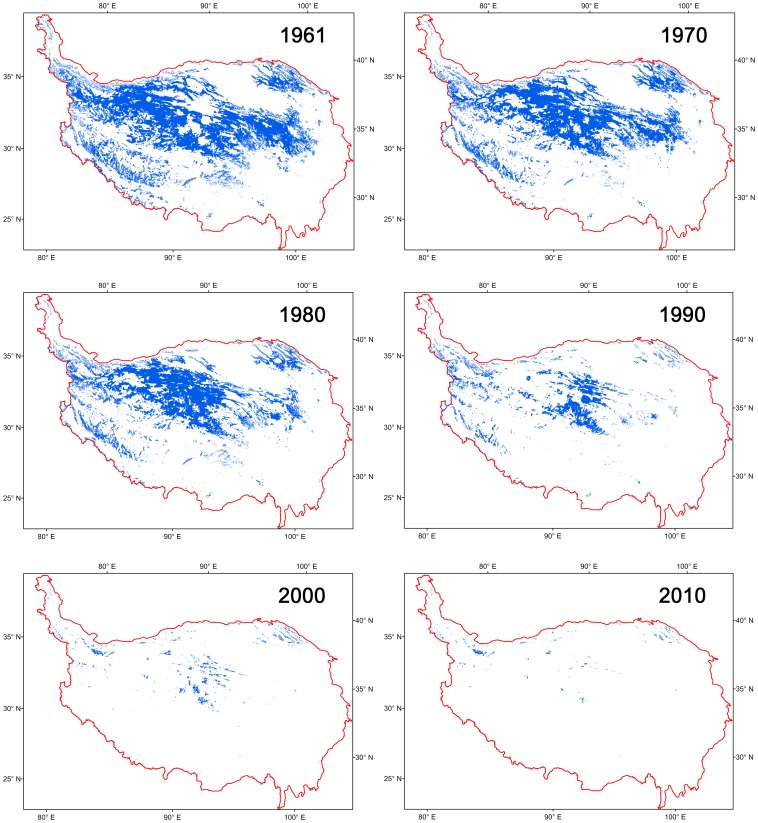
The change of the −6°C temperature zone in the TP in the characteristic years from 1961 to 2010.

To distinguish the colder temperature zones from the warmer zones, the colder annual mean temperature zones were shown in navy blue colours (Fig. 13). The cumulative temperature zones below −6°C were 648.86 thousand sq km in 1961, accounting for more than 1/4 of the total area of the TP. From 1961 to 2010, however, these areas had shrunk 636.22 thousand sq km and accounted for less than one per cent (0.5%) of the final total, a similar trend as that shown for the −6°C temperature zone (Fig. 14). The warmer temperature zones, in the Qaidam Basin and the southeast of the TP in the Brahmaputra valleys, under the same categorizing criteria, had expanded correspondingly. Obvious changes could also be found in the vicinity of Qinghai Lake, and the trends of these expansions were remarkable. These regions have flat terrain (average altitudes are 3000 m asl.), and with climate warming, the absolute surface mean temperature of the regions had increased almost 2°C during the last 50 years. The shifts of the enlarged temperature zones (warm red colours) and the shrunken zones (deep blue colours) demonstrated that the TP has experienced a significant warming period during the last 50 years. The changes of these temperature zones in the horizontal dimension were the results of vertical elevation of the isotherms. Spatial analyses of the changes can provide further understanding of the detailed warming differences in the spatial dimension.

#### Vertical changes

**Figure 15:**
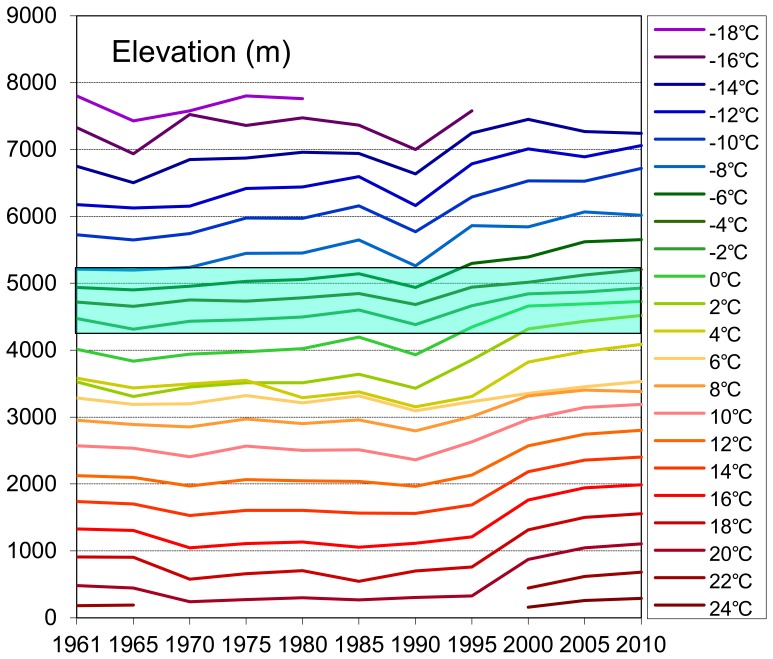
The mean elevations of each temperature zones in the characteristic years. The blue belt is the mean range of snowline/ELA of glaciers in the TP. The range is about 4200–5200 m asl. on average.

The shifts of isotherms and temperature zones are the most direct manifestations of climate change. With the increase in surface air temperatures, isotherms will be vertically elevated, and the temperature zones will change simultaneously. To further examine the trends of climate changes in recent years, especially in the vertical dimension, the mean elevations of each temperature zone in the characteristic years were calculated (Fig. 15).

The trends of the average elevations of the temperature zones were much smoother in the middle elevations of the TP (approximately 2000 to 4000 m asl.) and exhibited only small fluctuations before 1990, as seen in the statistical results in Fig. 15. The small uplift zones in the middle of the TP, e.g., the 6°C zones, had ascended only slightly, an average of 3287 to 3529 m asl. from 1961–2010. Nevertheless, the colder temperature zones on the top of the TP (shown in deep blue colours) and the warmer temperature zones on the bottom of the TP (in red colours) had increased prominently, especially over the past 20 years. For the elevated zones, the −12 to −6°C (above 5000 m asl.) zones were remarkably elevated. The elevations of the virtual isotherms are convincing evidence for global warming, and they displayed a gradual vertical elevation along with the warming. A representative ascending temperature zone is the 0°C zone, which ascended from 4014 to 4728 m asl. at an average rate of 14.3 m per year during from 1961–2010. On the whole, there was an abrupt significant increase in the elevation amplitudes since the beginning of 1990s. We hypothesise that an accelerated period of warming has occurred over the past two decades, but further proof and discussion are needed.

## Discussion

Temperature is a critical factor of climate change. Because the TP is a large and unique alpine climate zone, its climate characteristics are very different from the surrounding low altitude areas at the same latitudes. Warming trends in different parts of the TP are also quite different. The search for early signals of temperature variation in the TP may be applicable to assessments of the environmental impacts of global warming in other high altitude regions. Although there is some consensus regarding the warming trends in the TP, the short observation periods of the meteorological stations (most of the stations in the TP were established in 1961 or later), their altitude limitations (there are very few stations above 5000 m asl.) and their uneven distribution, especially in the sparse northwestern TP, have limited comprehensive research of the temperature variation in the TP. In this paper, we used the temperature zone as our analysis perspective and examined the changes in these zones. The calculated results of the GTEM demonstrated that the TP has undergone robust warming from 1961–2010. The temperature changes in the spatial dimension can be clearly examined by the GTEM. In the following section, several relevant issues are briefly discussed. We hope that our perspectives, which are supported by our analyses, will generate further discussion on this subject.

### Warming Trends

While the primary manifestations of climate change are physical in nature, such as alterations in temperature and precipitation, rising sea levels, and the increased frequency of extreme weather events, global change is a persistent and fluctuating phenomenon with significant uncertainty and shows the retreat, convolution, acceleration and deterioration of its effects [9], [41], [60]. During a short climatic period, however, change is always dominated by one type of climate regime, e.g., warming or cooling [3], [61]. The consensus is that global warming has been accelerating since the 1910s, and the trend of this warming is still an uncertainty under different development patterns and greenhouse gas emission scenarios [1], [60], [62]. As temperature variation is a primary manifestation of climate change, this paper has just discussed the temperature trends from the perspective of the temperature zone in the high elevation TP over the past 50 years and explicitly analysed the warming trend differences in the temporal-spatial dimensions with the GTEM using meteorological station records. The statistical results show that the curve of the GTEM has moved forward and that the gaps between adjacent curves have become larger in the TP, especially in the last 20 years of our monitoring period (Fig. 9). This phenomenon shows that the TP has undergone a significant warming period in recent decades and that this trend has gradually accelerated. This conclusion is consistent with previous studies of the TP based on surface temperature analyses, e.g., [27], [43], [44], [63]. Further analyses by the GTEM showed that the −6°C and −4°C temperature zones were the most significantly decreased zones and had fallen to 499.44 and 454.26 thousand sq km from 1961 to 2010 at average rates of 25.1% and 11.7% every 5 years, respectively. These regions were located in the northwestern and central TP and at high elevations. The analyses in the vertical dimension showed that, with the increase in the isotherms under global warming, the mean elevations of each temperature zone had increased at different rates. The significantly elevated zones were on the higher and lower elevations of the TP, while trends of the zones in the middle elevations of the TP were much more moderate (Fig. 15). Considering the distribution map of the temperature zone, the most significant altered regions were located in the northwestern and central TP and in several low altitude areas, e.g., the Brahmaputra valley, the Qaidam Basin and the vicinity of Qinghai Lake.

In this paper, our analysis was inspired by the isotherms and based on the perspective of temperature zone changes. The isotherms were elevated, and the temperature zones changed simultaneously due to global warming. Similar changes were also observed in other climate indicators, e.g., the snowline and treeline. Global warming has also caused shifts in the snowline, treeline, species composition and land cover in alpine regions because of altered climate regimes.

As the cryosphere is an amplifier of global warming, the equilibrium line altitude (ELA) or snowline has been elevated more significantly in response to rising temperature and the elevation of isotherms, with glacier shrinking being particularly evident in the Alps and Central Asia [32], [64]. The average altitude of the snowline/ELA in the TP reported in the literature is approximately 4200 to 5200 m asl. [9], [15], [32]. Based on the GTEM, this altitude spectrum was in the range of the −6 to −2°C temperature zones, and more importantly, the mean elevations of these zones were increasing with climate warming, especially over the last 20 years (Fig. 15). The outcomes are consistent with previous studies based on ELA monitoring. One such study, conducted by Vincent et al. [12], compared two mountainous regions of the world and concluded that alpine glaciers are strongly influenced by air temperature increases behind the elevated ELA in the Alps. In the TP and its surroundings, there are approximately 46300 glaciers with a total glacial area of approximately 59400 sq km and total volume of approximately 5600 cu km [32]. Because the TP contains considerable proportions of the glaciers in Asia, its changes are more representative as a way to monitor the effects of climate change. Yao et al. [32] found that glacial retreat in the TP and its surroundings has been a characteristic of the area since the 1960s and has intensified in the past 10 years. With the retreat, most of the snowlines of the glaciers had been elevated, and the retreat had caused an increase of more than 5.5% in river runoff from the plateau; simultaneously, glacial retreat had also caused rising lake levels in areas with large glacier coverage. Tobias [13] and Kutuzov [14] used remote sensing data to investigate the changes to glaciers and found that the temperature increases are two times higher than the global average in northern Tien Shan and very small increases in average precipitation were the main reasons for the snowline rise. Ding et al. [65] showed that more than 80% of glaciers in western China have retreated, losing 4.5% of their combined area, and that the mean ELA has become prominently elevated. Similar results were obtained by Li et al. [15] in an examination of the ELA of the Hailuogou glacier on Mt. Gongga (7556 m asl.), east of the TP; the data show that the absolute retreat ELA of the Hailuogou glacier since the 1930s has amounted to 300 m. The ELA is generally considered to respond positively to temperature and negatively to precipitation [66], [67], [68]. As a critical indicator of climate change, the ELA/snowline provides sufficiently robust proof of the process and further supports the conclusions from the GTEM. However, these observations are nearly sparsely located in the gigantic TP, and the observation periods are very short; it is difficult to detect climate change differences in the spatial dimension, even with remote sensing data, as they just cover a short observation period.

From the perspective of the treeline and ecotone, shifts of species towards the poles or to higher altitudes have been reported in response to recent global warming trends [16], [17], [18]. In general, the warmer current climate translates into better conditions for the recruitment and growth of trees at the upper limits of their ranges and facilitates the upward expansion of their population, while at lower limits it causes declines in growth and regeneration, resulting in the replacement of species by trees more suited to warmer climates [19], [23]. These trends are quite significant and are relatively easy to observe in alpine regions by comparisons with historic photographs. Baker and Moseley [20] used historic climate data and repeated photographs to assess alpine treeline and glacial recession in the southeastern TP (Yunnan province). These authors found that the warming rate in the most recent two decades was 0.06°C/year and that the warming was causing the retreat of glaciers and the vertical advance of the alpine treeline. Chen et al. [69] tracked the five travels of Ernest Henry Wilson (1876–1930) in western China from 1899 to 1918, then repeated the photos he had taken in his travels and compared them with the originals. The authors found that there was an obvious warming trend over the past 100 years, not only in specific areas but throughout all of Western China. Of the photos, 39 indicated that the treeline had elevated and that the vegetation had changed to suit the altered conditions. These comparative studies further support the results of our analyses based on the GTEM in the paper. As photographic technology is relatively new and long-term document photos are so rare, however, there is a limit to the power of comparative studies in this field.

The existing evidence suggests that global warming is driving the shrinkage of mountain glaciers and melting of permafrost, along with shifts in climate belts or species ranges poleward and towards higher elevations [21], [22], [23]. To confirm these shifts of climate belts and species redistributions under global warming, Hughes [24] studied the Australian continent, a remarkably flat region: 99% of the land area is less than 1000 m asl., and few summits exceed 2000 m asl., with the highest peak, Mt. Kosciuszko, at only 2228 m asl. As there is a lack of topographic relief, there is a limit on the ability of many species to shift to higher elevations as temperatures increase. Therefore, keeping pace with shifting climate zones requires long overland journeys to new geographic areas for the majority of species. This situation can be considered to be an exceptional example in the GTEM because when a region has a narrow spectrum of altitude, the GTEM curve will stay invariable with warming. Therefore, high elevation mountainous areas are always refuges for species sensitive to global warming [70], [71]. In the TP, with its wide spectrum of elevation and vertical zones, relevant research has demonstrated that species redistribution is highly associated with the magnitudes of the warming trend and precipitation conditions [20], [69]. However, the shifts in species ranges are due to the comprehensive effects of climate change, and it is difficult to attribute species migration to temperature increase alone. As a manifestation of global change, however, species shifts should be integrated with temperature data in comparative studies. Similar evidence has been reported in studies of climate change-induced land use cover change (LUCC) in high altitude mountain regions [25], [41]. Furthermore, these changed land use patterns could lead to changes in livelihoods [72], [73]. The same trends have been reported in different mountainous areas, e.g., the Andes [9], [26]. In the TP, Byg and Salick [41] considered morality and lifestyle to be the other main impact factors of LUCC besides climate warming. In addition to the climate factors, other anthropogenic factors, e.g., fire, should not be neglected [20]. Overgrazing and intensive human activity can also aggravate the deteriorating trends of LUCC [38], [40], [69].

In conclusion, there has been a robust warming period in the TP, especially in recent decades, and our GTEM results are consistent with previous relevant research related to temperature zone changes in the region. Most of these observational studies are short-term case studies, scattered widely over the large area of the TP, making it difficult to examine the warming trend differences in the spatial dimension; however, these studies cover different indicators of global warming to provide concrete proof of the temperature zone changes in the TP and further support our conclusions based on the GTEM in this paper. The integration of experimental and observational data with statistical meteorological records is vital for a synthesised analysis.

### Warming Trends with Temporal Scales

**Figure 16:**
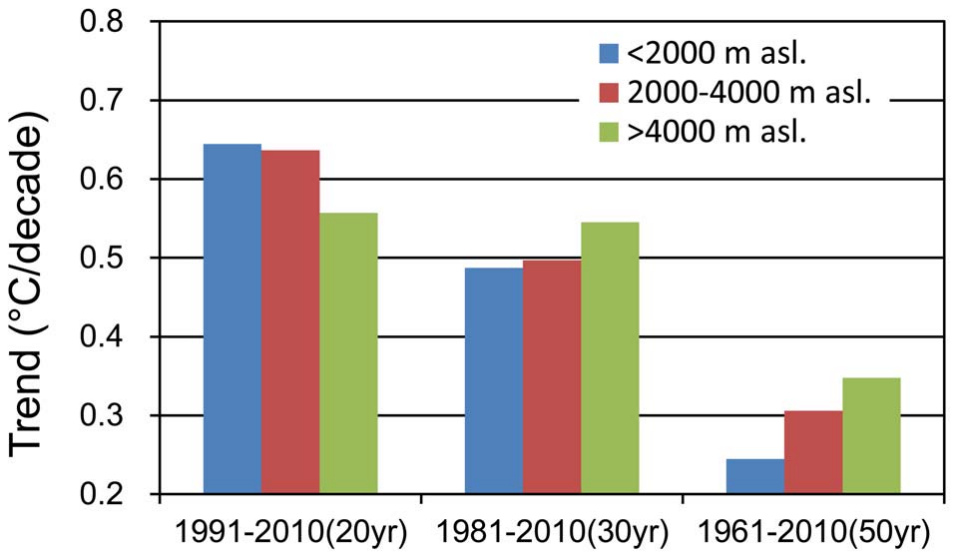
The average warming trends over the past 20, 30 and 50 years in different elevation zones in the TP and its surroundings.

It has been demonstrated, based on the GTEM using the surface annual mean temperature, that the TP experienced a significant warming process over the monitoring period from 1961–2010 and that the warming trend has accelerated, especially in the recent 20 years. To compare this accelerating process on different temporal scales and further analyse the differences in seasonal warming trends, we calculated the warming trends of each meteorological station during different periods. To examine the differences in the spatial dimension, we divided the 144 stations into 3 elevation zones at 2000 m intervals, i.e., 0–2000 m asl., 2000–4000 m asl. and above 4000 m asl. Warming trends over the past 20, 30 and 50 years for the different elevation zones were calculated (Fig. 16).

The statistical results showed that the warming trends in the TP and its surroundings have accelerated from 1961–2010. The mean warming trends for these 50 years were 0.24, 0.31 and 0.35°C/decade at lower, middle and higher elevations, respectively. However, in the past 30 years, the warming rates have increased to 0.49, 0.5 and 0.55 °C/decade, respectively. In the last 20 years, the TP has experienced an even more accelerated warming period, and the warming trends have increased to 0.644, 0.637 and 0.557°C/decade, respectively. These statistical results are consistent with the conclusions in the GTEM that the TP had experienced statistically significant warming over the past 50 years and that the warming trend was more prominent in the past 20 years (Fig. 15). In Fig. 15, the smoothly fluctuating elevation lines have significant increased since 1990, and their amplitudes have been increasing greatly year by year. Within the statistical ranges of the paper, the 22°C and 24°C temperature zones had appeared by the end of the study period (the dark red lines at the bottom of the figure). Similar conclusions can be found in several papers, e.g., Liu and Chen [44], Liu et al. [43] and Kang et al. [7]. Using 97 stations located above 2000 m asl. on the TP, Liu and Chen [44] found that the TP had experienced a warming period and that this warming had accelerated from the late 1990s. After analysing the trends of daily and monthly maximum and minimum surface air temperatures, the accelerated warming trends, especially since the 1990s, were confirmed [43]. The accelerated warming trend demonstrated that the global warming in the TP was significant over the past two decades. The changes of in the temperature rates demonstrate the warming trends in the TP and simultaneously support the results of the GTEM.

**Figure 17:**
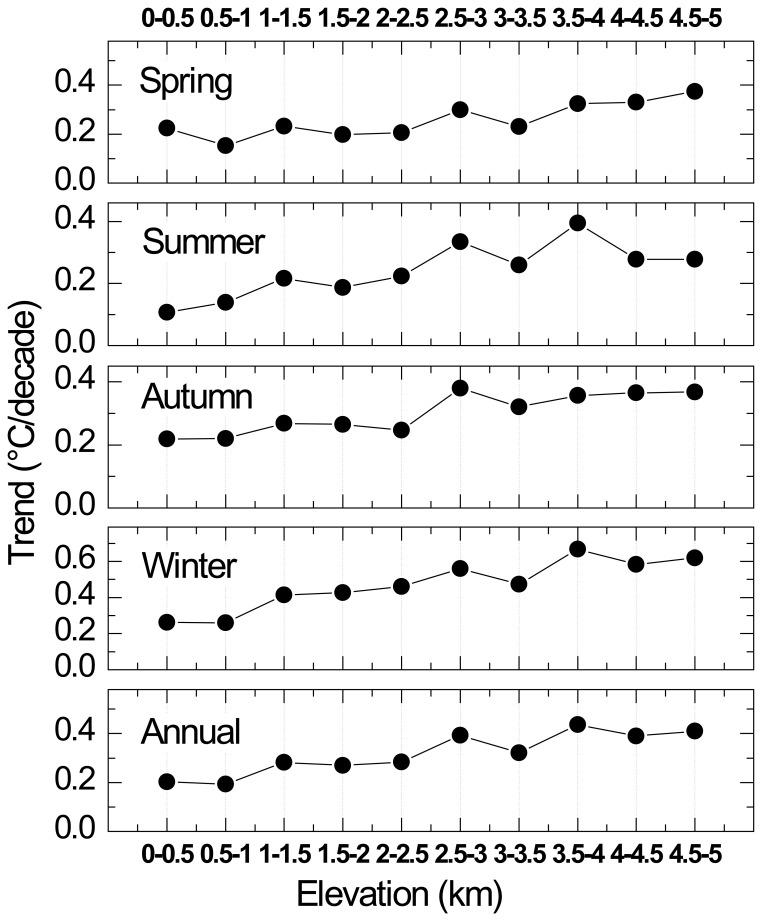
The mean linear warming trends of different seasons in each of the 500-m interval elevation zones during the period 1961–2010 in the TP and its surroundings.

Because it used data from annual mean temperature records, the GTEM neglected seasonal warming differences. We used the monthly surface mean temperature records as seasonal analysis data and calculated the warming trends for each meteorological station. To compare the trends between different seasons, we defined spring as March to May, summer as June to August, autumn as September to November and winter as December to February of the following year. The elevation was divided into 10 subzones at 500-m intervals to monitor the differences in the vertical dimension. In every zone, the mean linear warming trend was calculated for each season (Fig. 17).

**Figure 18:**
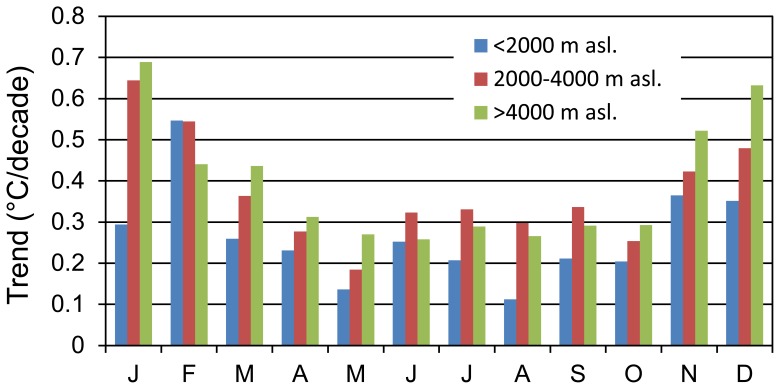
The monthly mean linear warming trends of the 2000-m intervals during the period 1961–2010 in the TP and its surroundings.

Comparing the trends in different seasons, the seasonal differences are quite clear, as seen in Fig. 17. The winter was the fastest warming season during the period from 1961–2010, with a mean warming trend of 0.473°C/decade. The maximum increase rate in winter was 0.668°C/decade at the 3.5–4 km asl. level. The season with the minimum increase was summer, with a warming trend of 0.242°C/decade. The annual temperature increase rate in the TP was 0.318°C/decade during the period 1961–2010, which closely approached the autumn rate of 0.301°C/decade. Further analysis of the monthly mean temperature increase rates classified into 2000-m intervals demonstrated that the warming trends were more prominent in January, February, November and December (Fig. 18). Climate warming was most robust in the winter months, followed by the spring and autumn months. The maximum increase rates were in January and December during winter, while the minimum rates were in May and August in the warmer seasons. These statistically updated analyses on the observational data further confirmed the conclusions from previous seasonal analyses [10], [27], [29], [44]. For example, Liu and Chen [44] examined monthly mean temperature trends at 197 stations in and surrounding the TP and detected more pronounced warming in winter. Wang et al. [63] analysed the annual mean surface temperatures increase rates averaged over 90 stations in the TP during 1960–2007 and obtained a value of 0.36°C/decade, higher than the 0.318°C/decade rate of this paper and double the previous estimate of Liu and Chen [44]. However, these authors reached the same conclusion that the warming trends were more significant in winter than in summer. Generally, these differences in rate were highly related to the choices of stations and research periods [7], [28].

While there is a consensus on the seasonal warming trends in recent years in the TP and its surroundings, the main factors and mechanisms underlying this phenomenon remain unclear. Duan and Wu [74] believed that the changes in cloud amount most likely contribute to the recent climate warming in the TP. As the TP is one of the most sensitive areas of snow feedback on Earth, snow/ice-albedo feedback is also considered to be one of the factors contributing to the recent warming in the TP [44]. Based on overall warming, other factors caused by human beings, e.g., anthropogenic greenhouse gas emissions and overgrazing induced land use changes, are generally considered to be the main causes of climate warming in the TP [1], [38], [39], [74]. Nevertheless, the exact reasons underlying the seasonal warming trend differences will require further investigation.

### Warming Trends with Elevations

Interestingly, an analysis of the warming trend differences in the vertical dimension suggests that the warming rates are increasing with the elevation in high altitude regions, a hypothesis known as the elevation dependency of climate warming [8], [9], [43], [75]. In our analyses, the GTEM results show that the −4°C, −6°C and −8°C temperature zones (zone mean elevations are 4860, 5176 and 5568 m asl., respectively, Fig. 15) have become significantly smaller during the past 50 years and especially in the past 20 years (Fig. 11). Similar trends can be found in the cumulative temperature zones below −6°C, −4°C, −2°C and 0°C (the mean elevations are above 4214 m asl., Fig. 12). As these zones are located in the higher elevations of the TP, their rapid reductions demonstrate that the top of the TP has undergone a robust warming period during the past decades. We tracked the changes in the −6°C temperature zone during the period from 1961–2010, and the zone nearly disappeared by the end of the monitoring period, as seen in the last three figures of Fig. 14. During this process, the mean elevation of the zone had increased by 715 m, from 4940 to 5655 m asl. Although the warmer temperature zones were concentrated in the narrow valleys and lower altitude regions of the TP, they still accounted for a large proportion of its total area (the areas above 0°C accounted for nearly 4/5 of the TP in 2010, Fig. 10). Accordingly, slight temperature increases would bring about a considerable enlargement of these temperature zones. Further analysis shows that the Qaidam Basin, the vicinity of Qinghai Lake, the southeast TP and the Brahmaputra valleys were the most remarkable warming regions under the same categorizing criteria in the figures (Fig. 13). Based on the GTEM, the warming trends were most significant at higher elevation of the TP and in several low altitude valley regions over the study period from 1961–2010. These results make the existence of the elevation dependency of climate warming in the TP questionable.

An operational method of analysing the problem is to divide the meteorological stations into different classes according to the elevation scale and calculate the statistical linear mean trend of each segment. To determine if the phenomenon is a universal or regional trend, however, the results must be compared over different temporal scales and regions.

**Figure 19:**
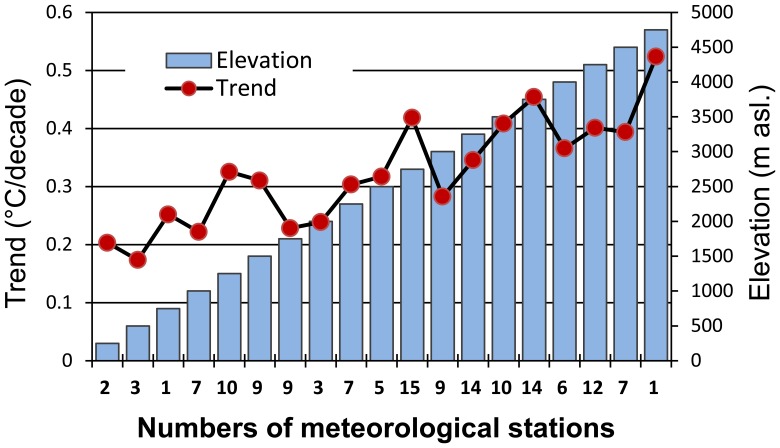
The mean warming trends of every elevation zones. The zones were divided by a 250-m interval based on the 144 meteorological stations in the TP and its surroundings during the period 1961–2010. The labels of the horizontal axis are the numbers of meteorological stations.

First, we divided the 144 stations into three elevation zones at 2000-m intervals. From the statistical results (Fig. 16), it is clear that the highest warming rate was at the top of the TP (above 4000 m asl.), the second was in the middle (2000–4000 m asl.) and the minimum was at the bottom (below 2000 m asl.) during the period from 1961–2010. Similar trends are displayed in Fig. 18 based on the monthly dataset analysis. In the majority of the months, the highest warming rates were found in the elevation zones above 4000 m asl. at the top of the TP, followed by the middle and the bottom. To confirm these results, further analyses using a higher elevation resolution of 500-m intervals were conducted and demonstrated that the statistical warming trends increased with elevation for each of the seasons (Fig. 17). Although there were some slight fluctuations between different elevations, the statistical upward trends were still remarkable. Warming trends in Fig. 17 also show that the elevation dependency of climate warming was much more obvious in winter than in summer. These outcomes are consistent with those of previous studies [8], [44]. To diminish the statistical errors resulting from low resolutions and monitor the subtle changes of the warming rates at different altitudes, we also calculated the mean linear warming rates for each of the elevation regions in 250-m intervals (Fig. 19).

These results showed that there was a statistically significant warming trend with the altitude increase in the TP during 1961–2010. Although there were great fluctuations across different elevation zones, warming rates still demonstrated an increasing trend with elevation. When compared with the trends of the 2000-m interval and the 500-m interval, the 250-m interval displayed greater variation. There are 13 stations with elevations below 1000 m asl. and only 1 station (Gerze station, No: 55248, 4763 m asl.) near 5000 m asl.; unfortunately, there were no stations located above 5000 m asl. The lack of a sufficient number of stations for the statistical calculations, especially in the western and central TP, led to large fluctuations in the calculated results. Despite these fluctuations, the statistical warming trends did increase with elevation, suggesting that an elevation dependency for climate warming in the TP was present during the period 1961–2010. These conclusions are consistent with previous studies by Liu and Chen [44], who examined the 97 stations located above 2000 m asl. in the TP from 1955 to 1996, and by Liu et al. [8], who monitored the 116 weather stations in and surrounding the eastern TP during 1961–2006.

However, further analysis showed that the tendency of the warming trend to increase with elevation in the TP has gradually declined in recent decades. As discussed above, an elevation dependency of climate warming did exist in the TP during the period 1961–2010, and the statistical results showed that the same trends were maintained during the period 1981–2010. During the accelerating warming process of the TP in recent decades, however, the gaps of the warming trends between the top, middle and bottom regions have narrowed (Fig. 16). Warming trends at the bottom of the TP accelerated more quickly in comparison with the other two areas until in the last 20 years (1991–2010), warming rates at the bottom had exceeded those of the middle and the top. This result demonstrates that, in the past 20 years, the bottom of the TP has experienced an accelerating warming process and that its warming rates have exceeded those of the middle and the top of the TP. In conclusion, the statistical warming rates were higher at the top of the TP from the perspective of entire study period, but quickly accelerated warming at the bottom of the region has altered this situation in the past 20 years. These results explain why the GTEM displays the Qaidam Basin, the vicinity of Qinghai Lake, the southeast of the TP and the Brahmaputra valleys as the most remarkable warming regions under the same categorizing criteria (Fig. 13).

The literature analysis also shows that there have been many previous studies doubting the existence of the elevation dependency of climate warming as a universal phenomenon but still considering it a regional trend due to the uncertainty behind its reasons and mechanisms [45], [46], [76], [77]. Vuille and Bradley [76] found that warming trends generally decreased with elevation increases in the tropical Andes but that the reason was unknown. Pepin and Seidel [77] found no statistical relationship between the magnitude of temperature trends and elevation in a global analysis of temperature trends for high elevation regions. You et al. [45] examined 11 indices of temperature extremes at 71 stations with elevations above 2000 m asl. in the eastern and central TP during 1961–2005 and found no significant correlations between the elevation and the trend magnitude of temperature extremes; they emphasised that the topographic type and degree of urbanisation were both factors with a strong influence on the analysis. The authors have presented similar conclusions in their subsequent research [46]. Even in approval studies, e.g., [8], [44], [75], there was no consensus explaining the phenomenon, and most of the conclusions were based on the statistics of meteorological records. Although an elevation dependency for climate warming existed in the TP and its surroundings during 1961–2010, this tendency has been gradually weakening, and both the GTEM and the warming trend-elevation statistics showed that the warming trends were more prominent at the bottom of the TP than at the top in the last 20 years. The elevation dependency of climate warming appears to be an exception or regional trend than a universal law, as surmised from meteorological records and case studies, and the reasons underlying the phenomenon are still unclear. However, the model remains a useful way to analyse warming trend differences in the vertical dimension in high altitude regions.

### Data Precision

The uncertainty and complexity of climate change are significant obstacles to its study, not only because it is difficult to simulate the trends of a large climate system under different topographical and regional conditions in high altitude mountainous regions but also because the extent of the impact, the magnitudes and adaptations on different levels vary depending on location [1], [78], [79]. However, climate change is a lengthy process that is constantly changing and adapting. To simulate the trends on different temporal scales and examine the warming trend differences in the spatial dimension, a Generalised Temperature zone Elevation Model (GTEM) has been established in this paper. Compared to the statistical results of the monthly datasets and the changes in relevant climate indicators observed in the literature, the GTEM was shown to be an effective method for analysing climate changes in the spatial dimension, especially in high altitude regions. As the annual mean temperature raster dataset was used in the model with 5 year-intervals and not with every year considered individually, there may be issues with the precision and generality of the data. Generally, the scale and precision of the data are the two basic sources of error [80]. In large-scale geo-data calculations, high data resolution may produce data redundancy and errors in the analysis process and may not always yield more believable results. Liao and Bai [81] discussed the errors of vector data changed to raster and emphasised that the data scale and land surface conditions (e.g., landscape or topography) were the key components of errors. Therefore, the choice of a suitable data scale and proper data criteria are crucial to the analysis. In this paper, when considering the amount and availability of the data, the annual mean temperature data were filtered by 5-year intervals, and the temperature zones were divided by 2°C intervals. Further analysis showed that the 5-year interval temperature data and the 2°C interval temperature zones could properly detect the trends of climate changes, and although we adopted these conventions, some details of more subtle temperature changes were inevitably ignored. The annual temperature data, when filtered by the 5-year intervals, may have included years with extremely high or low values. The outlier in 1995 demonstrates this situation. Additionally, unnecessarily high resolution (e.g., 1°C intervals) would have divided the temperature zones into very narrow belts with corresponding elevation gaps smaller than those of the 2°C intervals, obscuring the changes caused by the warming process. Further analysis showed that several 2°C intervals displayed inconclusive patterns (in Fig. 11, the area of the −4°C temperature zone increased before 1995 but declined afterwards, and in Fig. 12, the areas of the −4°C–0°C zones increased before 1995 but declined afterwards). These 2°C intervals, in effect, summarised two 1°C interval temperature zones with independent increase/decrease rates. However, the overall trends of warming manifested in the GTEM and the accompanying analyses demonstrated that the model was an effective way to analyse warming at different temporal scales and spatial dimensions. Further studies may utilise different data resolutions and compare the precision of different datasets to improve the analyses.

### Conclusions

According to analyses discussed above, we draw the following conclusions:

1. The GTEM model is an effective method for illustrating the distribution of temperature and can depict changes both in the temporal and spatial dimensions. The forward moving distribution curve of the temperature zones demonstrated that the TP has undergone a robust warming period from 1961–2010, especially in the past 20 years, and the mean warming rate over this period was 0.318°C/decade. Monthly data analyses showed that climate warming was most prominent in the winter months, followed by the spring, autumn, and Summer.
2. In terms of the spatial changes, the most reduced temperature zones were the −6°C and −4°C zones, which decreased 499.44 and 454.26 thousand sq km from 1961 to 2010 at average rates of 25.1% and 11.7% every 5 years, respectively. These quickly shrinking zones were located in the northwestern and central TP. Based on the GTEM, the warming trends were more prominent at the top of the TP and in several low altitude valley regions during 1961–2010, especially in the last 20 years.
3. An elevation dependency of climate warming in the TP did exist during the period 1961–2010, but the tendency has gradually weakened over recent decades due to the more rapid warming rate at the bottom than in the middle or at the top of the TP during 1991–2010. This result caused the GTEM to identify the Qaidam Basin, the vicinity of Qinghai Lake, the southeast of the TP and the Brahmaputra valleys as the most remarkable warming regions under the same categorizing criteria.
4. Comparison with relevant studies on other indicators of climate change in the literature, i.e., the snowline/ELA, treeline, species and LUCC, provided concrete support for the results of this paper.

## Supporting Information

Table S1 **The increased amounts and rates of each cumulative temperature zones.**
(DOCX)
