# Supplementary material for: Spatio-Temporal Characteristics of Global Warming in the Tibetan Plateau during the Last 50 Years Based on a Generalised Temperature Zone - Elevation Model
Source: PLoS One. 2013 Apr 2;8(4):e60044. doi: 10.1371/journal.pone.0060044 (PMC3615011; doi:10.1371/journal.pone.0060044)
Supplement: Table S1 — The increased amounts and rates of each cumulative temperature zones. (DOCX) [file pone.0060044.s001.docx]

**Table S1. The increased amounts and rates of each cumulative temperature zones**

| **Area / sq km** | **1961** | **1990** | **2010** | **increase amount from 1961 to 2010** | **increase amount from 1990 to 2010** | **increase rate / %** |
| --- | --- | --- | --- | --- | --- | --- |
| **Sum** | 2,571,577.8 | 2,571,582.1 | 2,582,573.2 |  |  |  |
| below -6ºC | 648,864.0 | 223,676.3 | 12,646.4 | -636,217.5 |  | -98.1 |
| below -4ºC | 1,209,817.8 | 863,363.7 | 119,342.7 | -1,090,475.1 |  | -90.1 |
| below -2ºC | 1,725,480.3 | 1,388,744.2 | 630,751.9 | -1,094,728.5 |  | -63.4 |
| below 0ºC | 2,105,118.5 | 1,801,814.3 | 1,167,480.9 |  | -634,333.5 | -30.1 |
| -4ºC to 0ºC | 1,456,254.6 | 1,578,138.1 | 1,154,834.4 |  | -423,303.6 | -29.1 |
| 0ºC to 4ºC | 706,114.0 | 936,961.8 | 1,479,677.5 |  | 542,715.6 | 76.9 |
| 0ºC to 6ºC | 753,685.4 | 1,057,576.4 | 1,726,410.4 |  | 668,833.9 | 88.7 |
| 0ºC to 8ºC | 781,039.3 | 1,100,534.4 | 1,828,112.2 |  | 727,577.8 | 93.2 |
| 0ºC to 10ºC | 799,866.5 | 1,125,749.1 | 1,870,421.0 |  | 744,671.9 | 93.1 |
| 0ºC to 12ºC | 813,651.1 | 1,142,655.2 | 1,894,073.2 |  | 751,418.0 | 92.4 |
| 0ºC to 14ºC | 823,318.7 | 1,154,701.2 | 1,909,771.6 |  | 755,070.4 | 91.7 |
| 0ºC to 16ºC | 832,053.9 | 1,164,730.8 | 1,921,574.2 |  | 756,843.4 | 91.0 |
| above 0ºC | 846,097.5 | 1,182,837.9 | 1,951,821.4 |  | 768,983.5 | 90.9 |
| **Average area above 0 ºC** | | | | | **714514.3** |  |
| **Average rate above 0 ºC** | | | | |  | **89.7** |
